# Retraction: Efficacy and safety of traditional chemotherapies for patients with ovarian neoplasm: a network meta-analysis

**DOI:** 10.18632/oncotarget.28834

**Published:** 2026-03-31

**Authors:** Lili Yang, Gongliang Guo, Liqun Sun, Chenhao Li, Haipeng Zhang

**Affiliations:** ^1^Department of Gynecology and Obstetrics, The First Hospital of Jilin University, Changchun, Jilin, China; ^2^Department of Cardiology, China Japan Union Hospital Jilin University, Changchun, Jilin, China; ^3^Outpatient Department of Pediatrics, The First Hospital of Jilin University, Changchun, Jilin, China; ^4^Department of Nephropathy, The First Hospital of Jilin University, Changchun, Jilin China; ^5^Department of Gynecology, The First Hospital of Jilin University, Changchun, Jilin, China

**This article has been retracted:** The authors of this article have requested a retraction due to the non-reproducibility of the published data. They noted that a potentially low number of cases in the original study has led them to doubt the validity of their analysis. All authors were in agreement with this request and the Editorial decision was made to retract the article. The authors and their institution (The First Hospital of Jilin University, Changchun, Jilin, China) were notified of that decision.

Original article: Oncotarget. 2017; 8:59867–59877. 59867-59877. https://doi.org/10.18632/oncotarget.16729

